# Co‐Design of a New Integrated Care Model With People Affected by Huntington's Disease: A Mixed Methods Study

**DOI:** 10.1111/hex.70584

**Published:** 2026-02-01

**Authors:** Sandra Bartolomeu Pires, Dorit Kunkel, Karine Manera, Nicholas Goodwin, Christopher Kipps, Mari Carmen Portillo

**Affiliations:** ^1^ NIHR Applied Research Collaboration Wessex, Southampton Science Park Innovation Centre Southampton UK; ^2^ School of Health Sciences University of Southampton Southampton UK; ^3^ Sydney School of Public Health The University of Sydney Sydney Australia; ^4^ Yong Loo Lin School of Medicine, Centre for Research in Health System Performance National University of Singapore Kent Ridge Singapore; ^5^ Clinical and Experimental Sciences, Faculty of Medicine University of Southampton Southampton UK; ^6^ Wessex Neurological Centre University Hospital Southampton NHS Foundation Trust Southampton UK

**Keywords:** care model, Huntington's disease, integrated care, micro‐level, mixed methods

## Abstract

**Background:**

People living with neurological conditions have needs that require an integrated care approach. Existing models of integrated care have often emphasized system structures but neglected the micro‐level interactions that matter most to people.

**Objectives:**

To develop a micro‐level model for integrated care that represents the care components most valued by people affected by Huntington's disease (HD).

**Methods:**

A mixed methods study with a co‐designed approach was delivered through three phases. This paper reports on the latest two, where interviews and workshops were conducted with people with lived experience of HD and professionals, from January to October 2024. Patient and public contributors were involved from project design to data interpretation.

**Results:**

Three themes were identified that position integrated care from the perspective of those affected by HD, representing these as the EC4Neuro model. Theme 1 identified the core components of micro‐level integrated care: expert knowledge, person‐ and family‐centred care, care coordination and continuity of care. Theme 2 underlined access inequities. Theme 3 highlighted people's responsibility to manage care without true agency to do so. The workshops prioritized strategies that enhance relational continuity between service users and providers. A tiered strategy was undertaken to support decision‐making towards improving person‐centred outcomes.

**Conclusions:**

EC4Neuro is the first integrated care model developed in HD. Its co‐designed approach with end users successfully embedded people's perspective to guide what needs to be achieved at the micro‐level. The EC4Neuro model offers prospective replication opportunities, particularly for stakeholders concerned with reducing access inequities and supporting relational continuity.

**Patient or Public Contribution:**

A group of 25 experts by lived experience of HD and other neurological disorders, co‐designed this research project, working with the researchers from conception of the studies to analysis and interpretation of the data.

## 1 Introduction

People living with neurological conditions require integrated care [1] but despite their complexities, neurological conditions have received less attention than other chronic diseases such as diabetes and asthma [2, 3, 4]. Neurological evidence has largely focused on prevalent conditions such as Parkinson's Disease (PD), often through clinician and researcher‐led approaches [5]. This leaves a knowledge gap on how best to support the needs of people living with rarer but complex neurological conditions, such as Huntington's disease (HD).

HD is a rare disorder characterized by progressive degeneration of the brain, with cognitive, psychiatric and motor symptoms [6]. In many individuals affected, changes start years before a clinical diagnosis of HD is formally given [7]. The disease currently has no cure. Each offspring of an affected parent has a 50% chance of inheriting the genetic abnormality [7]. Although classified as a rare condition, when a family is affected, HD is prevalent across generations.

People living with HD experience fragmented care, geographical inequalities in accessing care and serious unmet needs, that plead for an integrated and tailored response [8, 9]. However, no integrated care programmes have been developed in HD, leaving no clear pathway to the development of improved care models [10].

The World Health Organization (WHO) advocates people‐centred and equitable health system reforms to ensure access to care regardless of social or demographic characteristics [11]. Yet, reports from the HD community consistently highlight limited access to appropriate medical, social and supportive services [8, 12, 13].

According to the WHO, services can only be considered integrated if experienced as such by service users [14]. However, person‐centred outcomes are rarely measured in integrated care programmes, that remain focused on measuring clinical outcomes [10, 11]. Existing models have focused predominantly on macro (health and social care systems, governance and policy) and meso (organizational relationships) levels, with little evidence on the micro level (individual and interpersonal interactions), needed to implement and evaluate care at the personal level [15].

Recent expert opinion [16] has challenged that perhaps integrated care programmes have not delivered on improving people's outcomes precisely because their focus remains on macro‐ and meso‐levels, developing institutional‐ or health professional‐led models. Instead, experts recommend a ‘people‐driven’ approach [16] to achieve effective care integration, which responds directly to people's needs and enables them power in decision‐making through co‐designing care improvements.

To explore this hypothesis, this project aimed to co‐design a micro‐level integrated care model that identified the aspects of care most valued by people affected by HD. The project objectives were to:

– Develop an integrated care model based on the perspective of people affected by HD; and
– Incorporate person‐centred outcomes into its design.

## 2 Methods

### 2.1 Project Design

**Figure 1:**
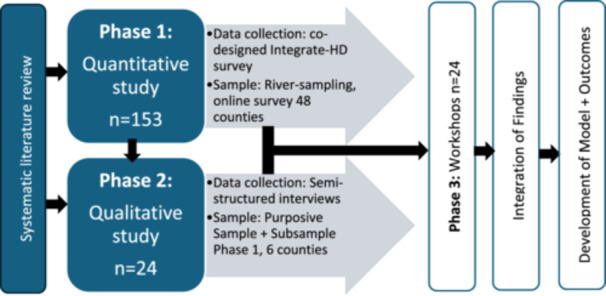
Integrate‐HD mixed methods sequential explanatory study design.

The Integrate‐HD project was developed following an international systematic review [10] that identified people with HD (PwHD) as underserved compared with other neurological conditions. Using a mixed methods sequential explanatory design [17], the project explored the various ways in which people affected by HD in England value how care should be integrated around their needs (Figure 1).

Table 1 summarizes prior phases of the project regarding their aims, methods and key findings. Phase 1, a cross‐sectional survey [8], revealed substantial unmet needs due to fragmented care; these findings recommended developing an HD‐specific integrated care model. Therefore, Phases 2 (interviews) and 3 (workshops) were conducted and are reported here, in line with Consolidated Criteria for Reporting Qualitative Studies (COREQ) guidelines [18].

**Table 1:** Integrate‐HD research phases.

Aim	Method	Key findings
Literature review [10]		
To establish if integrated care improves outcomes for people living with PD, Multiple Sclerosis (MS) and HD. To determine what characterizes a successful integrated care intervention.	Systematic literature review of multisectoral integrated care interventions undertaken in people living with PD, MS and HD. Strength of evidence rated for the different outcomes identified.	15 articles included but none on HD. Integrated care improved people's access to resources and reduced patients' depression. Few programmes considered caregivers' outcomes, reporting no difference or even worsening. Mismatch between people's needs and outcomes measured. Successful programmes were characterized by expert knowledge, multisectoral care coordination, care continuity and a person‐centred approach.
Phase 1 [8]		
To understand if current care provision meets the complex needs of people living with HD in England. To assess service users perceived need for integrated care.	Co‐designed cross‐sectional survey capturing the experiences of people living with HD in England. Quantitative analysis in SPSS (version 29) and qualitative analysis in NVivo (version 14).	153 people included. 65% of respondents rated their care as very poor, poor, or expressed a neutral opinion. Carers reported the lowest scores. People with access to a care coordinator reported improved care experiences, but only 19% of people had access. GPs and social workers were involved in care but were not knowledgeable. Top care priorities identified: mental health, family needs, financial and social support.

A pragmatist paradigm oriented to people's experiences was adopted, focused on practical solutions to real‐life problems [19, 20]. Accordingly, the research used a co‐design approach [21], combining participatory methods with patient and public involvement.

### 2.2 Patient and Public Involvement and Engagement (PPIE)

A PPIE group of 25 experts by experience of HD and other neurological diseases were recruited for this project, to comment on the potential impact of the HD model in the wider neurological field. The group's contribution to earlier work is reported elsewhere [8, 10]. This section describes examples of the group's co‐designing influence in Phases 2 and 3, while full PPIE work will be presented in a separate manuscript.

In terms of design, four contributors reviewed and refined the ethics application, co‐designed the interview guide, adding key questions to users, such as: ‘What is most important to you about your care right now?/What would make a real difference to you?’. These shifted data collection towards person‐centred care. Responding to concerns about participants' vulnerability, the protocol was amended to provide interview questions in advance, reducing anxiety and enabling fuller participation from cognitively impaired participants.

In terms of delivery, contributors supported recruitment, including one contributor joining a researcher on BBC Radio Shropshire to promote the study [22]. Targeting a county with recruitment challenges promoted inclusivity.

Four contributors also engaged in the early stages of analysis, inductively coding small transcript samples. This followed best practice for PPIE in qualitative research to reduce bias in interpretation from a clinical perspective [23]. In addition, an online workshop brought four contributors and a researcher together to refine the coding framework and discuss preliminary themes. This led to key distinctions, such as separating ‘expert knowledge’ and ‘upskilling knowledge’ and emphasized the broader relevance of the findings beyond HD, with potential wider implications for neurological care.

### 2.3 Participant Recruitment

Eligible participants were adults, both service users (SUs) and providers (SPs), living in England and fluent in English (non‐English speakers were excluded due to budget restrictions). Recruitment drew on a subsample from Phase 1 (from participants that had consented to be invited), and a purposive homogeneous sample that accounted for participant's county of residence and HD category. For Phase 2, six counties were selected based on their integration level (higher, lower and unknown), analyzed in the survey [8]. For Phase 3, the sample meant to be geographically diverse. A purposive homogeneous sample focused recruitment strategies in reaching people with different perspectives, such as people gene positive asymptomatic and people at risk of HD.

SUs included people at risk of HD, those diagnosed (clinically manifest or asymptomatic), informal caregivers and former informal caregivers. SPs included healthcare professionals (medics, nurses and allied health professionals), managers, academics, social workers, local authority and voluntary sector professionals, supporting PwHD.

Recruitment followed a community‐based approach, in collaboration with HD organizations, including the Huntington's Disease Association England & Wales. Advertising materials were shared through routes ethically approved. Individuals contacted the researchers, who clarified questions, confirmed eligibility and obtained written consent. Recruitment ran from January to October 2024.

### 2.4 Data Collection and Analysis

Data collection was conducted remotely: interviews took place via Microsoft Teams or telephone, and workshops via Microsoft Teams.

Phase 2 specifically aimed to explore integrated care from the perspective of service users and service providers involved in HD care. Therefore, interviews explored people's experiences with health and care services and interventions to promote integration.

One researcher conducted all the interviews (S.B.P.) following the interview guide co‐designed with PPIE. Four researchers trained in qualitative methodology (S.B.P., D.K., K.M. and M.C.P.) conducted the analysis. Interviews were transcribed and analyzed using NVivo (version 15), by applying the Framework Method [24], with the detailed guidance provided by Gale et al. [25]. The four researchers and four PPIE contributors independently coded three transcripts. Using an inductive‐deductive approach, researchers refined the analytical framework (codebook in Supporting Information S1: Table 1), after which one researcher (S.B.P.) coded the full data set and charted results into matrices in Microsoft Excel. The team (S.B.P., D.K. and M.C.P.) identified patterns, similarities and differences, to generate themes aligned with the study objectives. To enhance trustworthiness [26, 27], preliminary findings were discussed at a PPIE workshop in March 2025.

Phase 3 specifically aimed to discuss and identify core person‐centred integrated care outcomes valued by people living with HD and identify the key interventions to achieve those. Therefore, the workshops focused on discussing and ranking outcomes and interventions for developing an HD integrated care model generated from prior study data (review, survey and interviews).

Workshops followed an adapted nominal group technique [28], designed with a methodological expert (K.M.) and PPIE contributors, to ensure the active voice of service users in group discussions with providers. Participants received study materials in advance to facilitate informed discussions. Workshops quantitative analysis, conducted by two researchers (S.B.P. and K.M.), followed McMillan's method [29], which considers the votes strength and popularity. Two lists (interventions and outcomes) were ranked anonymously by importance. Scores were analyzed in Microsoft Excel: lower mean scores indicated higher importance (Rank 1 being most important), while vote frequency captured how often items were selected. Results are reported here to highlight participants' perspectives on what defines an HD integrated care model and the outcomes that best reflect whether care is experienced as integrated.

### 2.5 Data Sharing

The interview data are openly available in PURE [30]. The workshops data are not shared for the risk of compromising ethical standards.

## 3 Results

Interviews were conducted between January and June 2024 with 24 participants (15 users and 9 providers). Workshops ran in October 2024 with 24 participants (13 users and 11 providers). Although 27 were recruited, 3 users withdrew due to sickness, anxiety and fears of negatively influencing the workshops. In total, 46% of the workshop's participants had been previously interviewed. No dyads were recruited. Participant characteristics are presented in Table 2. Approximately 13% of participants were not British.

**Table 2:** Participant characteristics (*N* = 48).

Characteristics	Interviews		Workshops	
	Users	Providers	Users	Providers
Gender				
Male	7	2	5	2
Female	8	7	8	9
Ethnicity				
White British	14	7	12	9
Other White	0	1	0	0
Asian or Black	1	1	1	2
Age				
25–34	2	1	0	1
35–44	0	2	1	2
45–54	2	1	1	4
55–64	7	4	9	3
≥ 65	4	1	2	1
Category				
Gene positive asymptomatic	4	—	2	—
Former informal carer	4	—	1	—
Informal carer	4	—	6	—
At risk	1	—	1	—
HD diagnosed	2	—	3	—
Public sector provider	—	6	—	5
Private sector provider	—	1	—	2
Third sector provider	—	2	—	4
Total per category	15	9	13	11
Total per phase	24		24	

### 3.1 Interview Results: User‐Led Integrated Care Characteristics

Three major themes (Table 3) were identified: (1) Core components of micro‐level integrated care, (2) Access inequities and (3) Responsibility without agency in care. Representative quotes are in Table 4.

**Table 3:** Themes, theme definitions and sub‐themes.

Theme	Definition	Sub‐theme
1. Core components of micro‐level integrated care	Characteristics that need to be systematically and simultaneously delivered for integrated care to be experienced at micro‐level.	Expert knowledge Person‐centred care Family‐centred care Coordination Continuity
2. Access inequities	People with complex care needs are the ones that mostly need support but are the ones least likely to get it.	People's vulnerability Services' response
3. Responsibility without agency in care	Onus of care is set on patients and families, without appropriate resources to manage it.	Advocacy, empowerment or workload? Choice or neglect?

**Table 4:** Themes and sub‐themes with representative quotes.

Theme	Quote
*Theme 1: Core components of micro‐level integrated care*	
Expert knowledge	But I did have counselling when [PwHD] was bad, you know, with his moods and doing silly things, and to be honest, I didn't get anything out of it (…) because they didn't understand Huntington's. (SU10)
	So, there's an example of with the right advice and approach the budget for social services is much less because they don't now have to pay for a secure unit for him. He's fixed by and large. (SP4)
Person‐centred care	‘Well, there's no point in me going [to the clinic] because they're only going to (…) look at my gait or whatever, and actually that's not how I'm suffering’. So sometimes he would disengage (…) (SU6)
	(…) Social Services is a call centre, so you flag your concerns, you make your request and then it goes in a holding, waiting system, and then it gets triaged, and they make phone calls rather than visits (…) Everything is much more remote and not individualized. (SP3)
Family‐centred care	(…) the only place [care home] that would take him [father], so got moved all the way across the other side of the country and obviously me, my brother and my sister were all fairly young. (…) I couldn't go and visit him anymore (…) (SU1)
	(…) the families need as much support as the patients, to be honest, because they're going through an awful lot (…) I think the families could do with more support, some kind of helpline, some kind of services for them as well. (SP5)
Coordination	(…) everything was working quite well for her up to [month] (…) nobody was invited to the meeting, there was just him [Continuing Health Care staff] and my daughter and her husband – and miraculously after having the disease for 11 years my daughter suddenly became well. She didn't need any care whatsoever; she was deemed to be perfectly healthy and didn't need any care; and so that package was cancelled. (SU7)
	(…) it's a huge burden for them to have to deal with (…) if you had one individual who oversaw the need to pass on information and link people together and kind of undertake those assessments and pass on that assessment information, that would make life an awful lot easier, wouldn't it? (SP9)
Continuity	No, I got fed up with it, with going [to the GP], and whoever was the sort of temporary doctor [GP] would just freak out every time (…) I sort of investigated one of the partners there (…) I spoke to him and I said, ‘Right, look, I want you.’ I said, ‘I don't want to be floating now’ (…) When I go into the GP and they put my notes up on the screen, so it's got a banner up there, you know saying I've got Huntington's (…) the nurses saw every time you'd phone in, ‘Oh, are you okay?’ which I appreciate (…) But then you're being risk managed (…) thanks to the team, with [HDA advisor] and everybody else saying, you know, ‘Don't float, let's try and get somebody specific as a GP’, thankfully, as a tick in the box, because you know, you will encounter lots of horror stories. (SU3)
	(…) even if they are under the Mental Health Act Section, they sometimes might be declined a bed. (….) you can't have a physical health hospital, general hospital, because it's not exactly that, and then psychiatrists say, ‘Well, this is Huntington's so it's not mental health so we're not getting involved’. So, there is sort of… sometimes we do get into situations where the patient finds it difficult to access help because of the lack of belonging (…) (SP1)
*Theme 2: Access Inequities*	
People's vulnerability	The patients with the most complex problems get the most fragmented service. (SU9)
	If people answer in the affirmative and say ‘yes, yes, yes, I'm fine’, yeah, they are going to lose access to money and they're going to lose access to services. And I think it's like shark infested waters (…) it's fraught with difficulty out there for people because it's so hard for them to navigate successfully to actually get the big prize of having a service which they should be entitled to anyway. They should be entitled to good care (…) to a seamless assessment that actually improves things and doesn't make things worse for them. (SP9)
Services' response	In honesty if I wasn't proactive about things nothing would happen I don't think so, no, I can't really think of anything positive. (SU4)
	It's frustrating because you would ideally want to get those services involved to prevent it reaching that crisis point and that seems to be a bit more difficult to achieve, yeah. (SP2)
*Theme 3: Responsibility without agency in care*	
Advocacy, empowerment or workload?	(…) I was lucky, because you know, as you can tell, I'm *compos mentis*, I can make my own argument, I can talk to somebody on the phone, I can talk technically and clearly, you know, so yeah…that was frustrating. But I was more than capable of doing that. (SU3)
	The difficulty comes with arguing with funders to provide adequate funding so we can staff adequately. It comes within fighting for resources for whether that's a wheelchair or a bed or an assessment or things like that. That's the struggle to us (…) being the Advocate for these residents (…) (SP8)
Choice or neglect?	he was home (…) it was supposed to be a Friday to a Monday leave and whatever. The social worker came around on the Friday morning, he was like ‘Yeah, I'm fine, I'm fine’. (…) half an hour chatting the social worker said to me, ‘Well, I think I'll close this case because there's no need to be here. Whatever.’ I'm like ‘No, no, no, no. Please don't. (…) ‘I'll close the case’ (…) later that night, again, he went into a massive psychosis and that so I ended up just calling the police. (…) he was too complex for everybody. And I think again, that's the problem. So they minimalized it because then they didn't have to deal with it. (SU6)
	(…) we think there's a lot in self‐neglecting patients, who are (…) in terrible trouble, but because when the social services come round, and sometimes mental health teams as well, and say, ‘How are you doing?’ and they go, ‘I'm fine’. And then they write back saying, ‘Patient has full capacity and has declined the service, goodbye’. And that doesn't seem right to me, that seems like a dereliction of duty, and duty of care (…) you can see the patient isn't fine, they might think they're fine, but they could be dead tomorrow because they don't know how to cross a road (…) And they go, ‘Well, they just said it's fine’. That's not enough, that's not a subtle enough argument. Sometimes when somebody says they're fine, you've got to be able to say, ‘Actually, no, you're not’. (SP6)

Abbreviations: GP, general practitioner; HDA, Huntington's Disease Association; PwHD, person with Huntington's disease; SP, service provider; SU, service user.

These themes reflect the care characteristics required in a HD integrated care model. The next section delves into each theme.

#### 3.1.1 Theme 1: Core Components of Micro‐Level Integrated Care

Five components were identified.

##### 3.1.1.1 Expert Knowledge

Effective HD expert teams were described as multidisciplinary, combining neurology and mental health expertise and acted as tertiary centres. Providers emphasized the need to upskill professionals outside the expert team, especially social and mental health professionals, who were frequently involved in HD care.

Interviewees perceived ill‐consequences from having varied professionals with limited HD knowledge involved in care: ‘He [psychiatrist] saw [PwHD] (…) and just said “You're stable. You don't need any mental health support” (…) we were in an absolute crisis point (…) She should never have been discharged (…) when she passed away (…) she was literally skin and bones’ (SU9). Participants suggested identifying and training HD link workers within such teams, with experts accessible for consultation. Providers stressed that with appropriate treatment and care, PwHD could improve, underlining the importance of having experts available.

##### 3.1.1.2 Person‐Centred Care

Quality of care was reported as multidimensional, accounting for people's bio‐psycho‐social and environmental needs in the development of care plans: ‘He really changed because he had stuff to do, he had some responsibility (…) He had a little job, voluntary work; she got him free gym membership (…)’. But participants observed that formal care mostly focused on meeting physiological needs: ‘(…) the [formal] carers just…currently they just go in for food and medication, and personal care. But it would be nice if there's more enjoyable things done with him, like going for walks’ (SU12).

Furthermore, bureaucracy was often perceived to obstruct person‐centeredness, for example, referral letters were required from the general practitioner (GP), rather than from the charity advisors, who reportedly knew the PwHD better (SP2).

##### 3.1.1.3 Family‐Centred Care

Only one family caregiver interviewed (SU13) reported having appointments with an HD nurse specialist, vastly praising its value. Participant reports highlighted how caregivers often neglected their own health, and that caregivers specific support was required.

Family support was raised particularly where participants held multiple roles, such as when a person was both at risk of HD and was also an informal caregiver for someone affected by HD. Participants emphasized the need for intergenerationally sensitive care, since HD affects people across different life stages. Individuals underlined their experiences of how children and young carers felt particularly unsupported, often precipitating family crisis: ‘She [social worker] said, “[PwHD] has to go into a home” (…) we had two young daughters at the time, and [PwHD] still wanted to be their mother’ (SU9).

##### 3.1.1.4 Coordination

Care coordination was understood as successful when professionals involved were accessible, knowledgeable, system literate and able to bridge between sectors. Care coordinator roles were described as performed by specialist nurses, occupational therapists and charity advisors. Benefits described were family advocacy, prevention of safeguarding failures and timely care. However, providers (SP5, 6, 8) warned that the capacity to coordinate care was undermined by workload and bureaucracy: ‘(…) the extra work that goes into making these proofs is ridiculous (…) for it to be immediately rejected (…)’ (SP8).

##### 3.1.1.5 Continuity

Participants reported a near‐even split between both positive and negative experiences of care continuity (references negative: 28, positive: 26).

Information continuity [31] relied on eHealth solutions (such as QR code based records) and, above all, professionals' familiarity with the patient. Often, relatives were the primary information source. Cross‐boundary and team continuity [31] was a major challenge, with transitions and referrals leading to fragmentation. Improvements came through care coordinators, named professionals, shared care agreements and established care pathways. Longitudinal continuity [31] was valued when participants could maintain the same GP or access long‐term care settings competent in HD care, even if this required longer wait or greater travel.

Flexible continuity [31] depended on staff expertise and users' capacity for self‐management, which declined as disease progressed. Therefore, relational continuity [31] was crucial in humanizing care, particularly given cognitive and behavioural changes: ‘Went to the dentist (…) She said (…) “if the receptionist rings you (…) and you're in a period of dysregulation, we don't want the receptionist to think, oh, she's an awkward person. We need the information on your file to say, you know, if you get that response, try calling her another day. It's not the person”. So, that was very joined‐up care’ (SU14).

Themes 2 and 3 highlight caveats requiring particular attention for the development of an integrated care model in HD.

#### 3.1.2 Theme 2: Access Inequities

Those most in need of an integrated care approach were the least likely to access it.

##### 3.1.2.1 People's Vulnerability

Vulnerability arose from psychiatric symptoms that impaired access, lack of HD knowledge among professionals involved and stigma.

Over half of participants (58%) reported delays in urgent responses, particularly for mental health crises: ‘(…) an urgent situation can go from urgent to quite seriously dire in that period of time, so it's leaving vulnerable people in some very unacceptable situations’ (SP3).

Vulnerability seemed heightened for those with psychiatric symptoms but minimal motor signs, suggesting stigma: ‘I actually do believe if he had lots of chorea, it would've been different’ (SU6).

##### 3.1.2.2 Services' Response

While people's vulnerability required a proactive response from services, participant experiences portraited services reactive responses, intervening mostly at crisis point. Exceptions were reported tough relative to charity advisers, that according to participants' reports, consistently provided proactive support.

Clinical reviews were considered as valuable as for other chronic diseases, helping prevent crises by identifying needs earlier, such as ordering equipment to prevent injuries: ‘I guess it's like I do for my [chronic lung disease] (…) if you could have something where you went every six months, you went to a specialist who understood the disease (…)’ (SU15).

#### 3.1.3 Theme 3: Responsibility Without Agency in Care

A significant component of the burden of living with HD came not from the disease itself but from systemic caring failures (Supporting Information S1: Table 1). The responsibility of care was set on patients and families, but the system failed to equip people with appropriate resources to have agency in their care.

##### 3.1.3.1 Advocacy, Empowerment or Workload?

Participants stressed the gap in empowerment: ‘(…) to enable people to be able to do those things, those things need to be there’ (SU14). Participants felt people were expected to manage their disease without adequate education or resources. Furthermore, self‐management was perceived especially difficult for symptomatic PwHD, facing cognitive and psychiatric decline.

Relatives described the frustration in educating professionals: ‘In fact, the geneticist, did she use the word «advocacy» for HD? “Go to the GP and be an advocate for HD”’ (SU15). But the need to advocate was also reported by HD providers, who often faced disbelief from non‐HD care services; ‘Fighting,’ ‘battling’ and ‘advocating’ terminology appeared in 60% of users and 56% of providers accounts. Advocacy was particularly reported at coordination points (referrals to therapies, respite, benefits, care packages).

##### 3.1.3.2 Choice or Neglect?

While some in earlier HD stages reported positive choice in service use, most advanced cases described false choice, with services reduced or denied under the guise of patient refusal. Participants described this as neglect or ‘dereliction of duty’ (SU6). Caregivers that had complained formally, reported being ignored, resorting to direct intervention: ‘“I've whisked him out, he's not safe” (…) and then with three or four days (…) they closed the home down’ (SU3).

Accountability was viewed as key to reducing neglect, through mechanisms such as HD institutional accreditation schemes, professional registration (e.g., with the Nursing and Midwifery Council), and external regulation (like Care Quality Commission). Yet participants felt that action only followed crises: ‘policy gets changed because something bloody awful happens. So probably someone will die in squalor and then it will be in the papers and everybody will say, “Something has to be done”’ (SP6).

### 3.2 Workshop Results: Prioritizing Change

This section reports on what to prioritize in the development and assessment of an HD integrated care model, based on the workshops conducted. The top‐10 ranked items for interventions and outcomes are presented in Table 5, Supporting Information and sub‐group analysis are detailed in Supporting Information S3: Appendix 1.

**Table 5:** Top 10 interventions and person‐centred outcomes ranked by participants in the Integrate‐HD workshops (analysis in Supporting Information S3: Appendix 1).

Interventions	Ranking	Outcomes
Care coordinator	1	Expert Huntington's disease team
HD hub/one‐stop‐shop multidisciplinary care (experts, including social worker)	2	Access to care
Mental health teams with an HD link member	3	Knowing who to contact
Appointments that are person‐centred (needs‐based)	4	Single contact
Caregivers' appointments (review caregivers' needs)	5	Response time
Social workers integrated in HD team	6	Quality of life
Regular appointments and follow‐ups	7	Caregivers' stress
HD National Institute for Health and Care Excellence (NICE) guidelines	8	Financial support
Caregivers training	9	Crisis episodes
Care settings fitting people's needs (home‐based, day‐ and long‐term care for adults with complex neurological needs)	10	Social interactions

Sub‐group comparison between service users and providers revealed consensus on the top 2 ranked interventions: a care coordinator and an HD hub. Interventions related to mental health teams accepting HD referrals and having a clear link with the expert HD team and/or care coordinator, were ranked in the top 5 by both users and providers. Differences in ranking highlighted that user participants weighed more importance on four interventions than providers: community resources, social worker with a family‐centred approach, 24‐h line to reach an expert, and community engagement/social activities connected to the HD community (bringing services closer to the community, e.g., activities with children at risk). Providers though privileged healthcare interventions, such as the creation of National Institute for Health and Care Excellence (NICE) guidelines, development of HD care responses (home‐based care, day‐care and long‐term care) and providing regular follow‐up appointments.

For integrated care outcomes, sub‐group comparison between service users and providers revealed consensus on the top 4 ranked outcomes, related to access to expertise and improved relational continuity. Differences in ranking highlighted that user participants weighed more importance on outcomes related to quality of life and family wellbeing, as well as outcomes related to wider determinants of health, such as financial and home support (shopping, housework, amongst others). While provider participants weighed more importance on healthcare‐related outcomes, such as crisis episodes.

### 3.3 Integration of Findings

As a mixed methods project with an explanatory design, the last phase of the Integrate‐HD project focused on interpreting how the qualitative findings complement and explain the quantitative findings, and what is the overall learning in response to the project objectives [17].

Qualitative data from the systematic review [10] identified four characteristics of integrated care models in diseases such as PD: expert knowledge, person‐centred, coordination and continuity. Integrate‐HD interviews, however, highlighted that family‐centred care is equally critical in HD, as care often involves both the PwHD, the informal caregivers and a ripple effect across generations. This finding explains the project's quantitative data survey [8], where family needs emerged as participants' top priority.

**Figure 2:**
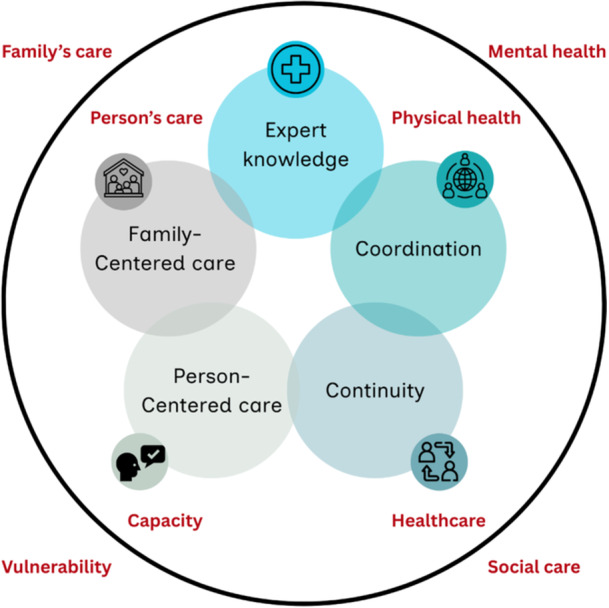
EC4Neuro model, with its integrated care components: *E*xpert knowledge, *C*oordination, *C*ontinuity, Person‐*C*entered and Family‐*C*entered care. Around, the most visible (inside the circle) and invisible (outside the circle) aspects of care.

The five core components—expertise, person‐centred care, family‐centred care, coordination and continuity—were further validated by Integrate‐HD workshops, where top‐ranked interventions and outcomes corresponded to these components (Supporting Information S2: Figure 2).

While the survey [8] identified unmet care needs, the interviews provided an in‐depth understanding of care fragmentation and vulnerability in HD. These insights suggest four ‘bridges’ needed towards care integration:

– Assessing a person's capacity and vulnerability to manage care.
– Identifying individual and familial care needs.
– Supporting physical and mental wellbeing.
– Establishing relational continuity across health and social care systems.

Figure 2 illustrates these core components and bridges, presenting the EC4Neuro model, co‐designed with people affected by HD, to achieve an integrated care experience at micro‐level.

Table 6 integrates the project data, aggregating the EC4Neuro core components with participants' priorities for change, highlighting strategies to improve care integration.

**Table 6:** Strategies to improve care integration aggregated according to the EC4Neuro model.

Tiers	Expert knowledge	Coordination	Continuity	Person‐centred care	Family‐centred care
Low	Professionals consult with nearest HD expert team. Inter‐professional HD education.	Social worker appointed and reachable. Identifiable HD expert teams.	Regular appointments and follow‐ups. Regular follow‐ups at the GP with referral to HD experts when needed. GP identifies and electronically records people at risk and caregivers.	**Appointments are person‐centred and needs‐based.** **Signposting to community resources.** Longer GP appointments (double slot). Care plan reviewed regularly. Non‐pharmacological therapies offered. Individual care plan.	**1:1 appointment for the informal carer** (with expert team or GP). Family kept updated and involved with the care plan.
Intermediate	**Social worker** integrated in expert team.	**Care coordinator** available.	**Mental health** team accessible and with HD link pin. Social workers hold cases for longer. Appointments with same GP.	Self‐referral system to expert team. Roadmap booklet/app/resource to help people navigate living with HD.	**Social worker** supports family across the lifespan. **Professionals involved with the HD community** (e.g., developing activities with children at risk). Caregivers training provided. Formal carers supporting with household tasks.
High	**24‐h expert helpline.** Expert multidisciplinary team available. Core team of GPs particularly trained and involved with PwHD. NICE guidelines development.	Raise public awareness. Upskill professional workforce. Increase funding to healthcare, social care and research.	Centralized electronic records between sectors. Patient passport/QR code/key medical history accessible.	Care settings fitting people's needs: home‐based care, day‐care and long‐term care for adults with complex neuro needs. Robust care pathway.	**HD hub** caring for people at risk to advanced stages with non‐pharma activities (including social worker). Informal caregivers considered part of the workforce (financial remuneration).

*Note:* Interventions are suggested from low to high resources required to implement that change in the English context. Service users' top‐ranked interventions are signalled in bold.

Abbreviations: GP, general practitioner; HD, Huntington's disease; NICE, National Institute for Health and Care Excellence; PwHD, person with Huntington's disease.

## 4 Discussion

To the team's knowledge, the Integrate‐HD research programme has developed the first model of integrated care for people affected by HD, guided by what matters most to them, in a co‐designed approach. The EC4Neuro model includes five integrated care components essential at user‐level: expert knowledge, person‐ and family‐centred care, care coordination and continuity of care. In addition, the project identified person‐centred outcomes, with emphasis on care that is provided by experts and promotes relational continuity.

### 4.1 Addressing Health Inequalities

Although its implementation remains to be tested, the EC4Neuro model draws on previous evidence [10] into the characteristics of integrated care programmes in prevalent neurological conditions. This research corroborates the importance of the four characteristics identified then [10]: expert knowledge, person‐centred care, coordination and continuity. Importantly, this study conducted in the context of a rare neurological disease, contributes to shape integrated care models with a fifth key characteristic: family‐centred care. The importance of family‐centred care had been mentioned in other rare diseases studies, such as in CONCORD [32], which suggested coordinated family‐centred appointments to improve communication, decision‐making and reduce travel burdens.

EC4Neuro differs from other models in prevalent neurological disorders such as PRIME [33] and iCARE‐PD [34], which emphasize self‐management. Due to HD‐related vulnerabilities, cognitive decline and psychiatric symptoms, self‐management is often unsustainable. Comparative studies have shown that PwHD experience more severe symptoms, lower mood and poorer quality of life than people with PD [35], highlighting the need for tailored care models that address these distinct predictors [35, 36].

Integrate‐HD interviews revealed that half the burden experienced by PwHD arises from extrinsic factors related to fragmented care or underserved needs. Similar challenges are reported in other rare diseases [37, 38], suggesting that integrated care must adopt a people‐driven approach to reduce health inequities. Future research should focus on identifying needs across rare neurological disorders and explore system‐level changes to improve care for these populations.

### 4.2 Integrated Care Impact at Micro‐Level

A key Integrate‐HD contribution is the assessment of care integration at micro‐level, an under‐researched area [10, 39]. Ranking exercises showed that person‐centred outcomes mirrored the EC4Neuro model components, validating the model from a service user perspective. From the person‐centred outcomes identified, access to an expert team was the primary outcome (Table 5) ranked by participants, reflecting difficulty in obtaining specialized care. Evidence from ultra‐rare disease contexts suggests that access to multidisciplinary ‘one‐stop’ clinics improve outcomes without increasing costs, although specific outcomes data remains limited [40].

EC4Neuro contributes with person‐centred outcomes for evaluating integrated care at the micro‐level. Compared to broader neurological studies, such as Spiers et al. [41] which emphasized personal comfort, autonomy and social participation, EC4Neuro captures outcomes across all five important integrated care components, likely due to its co‐designed inclusive approach.

Top‐ranked outcomes agreed between service users and providers, such as single‐point of access, corroborated the importance of relational continuity for people with mental health needs [42]. But the study also makes a novel contribution in showing divergency between users and professionals. While service providers prioritized outcomes related to health and care utilization, service users prioritized outcomes related to the wider determinants of health. These differences underline the importance of co‐designing integrated care programmes with a person‐driven approach.

Acknowledging the missing evidence in assessing integrated care interventions from service users' perspectives [10, 11], this study advances knowledge in providing co‐designed person‐centred outcomes. These results should be explored in a wider sample to facilitate the evaluation of future integrated care programmes at micro‐level.

### 4.3 Implications

The current study provides practical guidance for service improvement from a user‐led perspective. Top priorities, identified in this project, have been tested in other chronic diseases, improving people's outcomes: assigning a care coordinator [43, 44, 45]; linking patients to multidisciplinary hubs [46, 47, 48]; and strengthening collaboration between teams through fostering linking roles [49, 50]. Such interventions could provide similar benefits if tested in people affected by HD and other rare neurological diseases. EC4Neuro offers a tiered strategy adaptable to different contexts, many of them at no added costs; for example, primary care practices might lack capacity to train teams extensively on HD but can improve continuity of care by offering the same GPs for PwHD.

Equally, EC4Neuro enables the co‐designing of services through role adaptation (e.g., social workers embedded in HD expert teams), multi‐sectoral pathway definition (e.g., coordination between expert and mental health teams), staff training (e.g., standardized protocols development) and outcomes monitoring (e.g., evaluate metrics that mirror important participant‐ranked outcomes).

At a macro‐level, EC4Neuro can support people‐driven system reforms by guiding decision‐ and policy‐makers to strategize changes that directly respond to people's needs. The model provides a framework of what matters most to service users, empowering them to co‐designing a system that delivers on what is important to people. For instance, the top‐ranked intervention, a care coordinator, highlights care inequities across Europe that require action. While in the Netherlands, PwHD have widely benefited from accessing a care coordinator for the last 10 years [51, 52], in England less than one quarter of people living with HD report access to a care coordinator, marked by post‐code lottery. These inequalities, reported in other neurological conditions in Europe [53, 54], emphasize the need for cross‐country knowledge mobilization where people‐driven approaches are applied to policy and practice [16].

A recent example of people‐driven policy is the implementation of Martha's Rule in England [55]; a patient safety initiative to support the early detection and escalation of deterioration by empowering patients, families, carers and staff to be listened to and request a rapid review from a different team. Marta's Rule [55] was prompted following the death of Martha, aged 13, which was considered potentially avoidable should her family's concerns had been responded to. With a co‐designed approach, the Integrate‐HD project raises evidence that ‘Something has to be done’ (SP6) to improve care integration in HD, suggesting a model that can support governments in how to deliver people‐driven change.

### 4.4 Challenges

Although further work is required to analyze the model's possible cost implications, previous studies in PD [56, 57] suggest that an integrated care approach could promote a more affordable care system in the future. While the evidence in HD is sparse, it suggests similar cost‐savings could be achieved [58, 59]. But implementing such a model comes not without challenges, from which funding is one of many.

The integrated care bridges suggested, such as establishing continuity across health and social care systems, require collaboration between services and sectors. Embedding social workers in HD expert teams would need an implementation plan that straightens collaborations between these sectors through interrelated domains [60]: motivation and purpose (e.g., vision), relationships and cultures (e.g., trust and professional cultures), resources and capabilities (e.g., such as funding, data sharing), governance and leadership (e.g., decision‐making and engagement) and external factors (e.g., national policy). Evaluating change in these complex domains is difficult, namely when trying to establish if an intervention improves health and social care integration [61]; therefore, future evaluation of the EC4Neuro model will present challenges. That is why having a clear vision about what is trying to be achieved is so important [62] and a realistic view on the amount of time it takes to achieve success [63]. In agreement with the WHO [14], the model's vision reflects the users' perspective on care integration as a beacon for change.

### 4.5 Strengths and Limitations

Integrate‐HD strengths include its co‐designed methodology, promoting inclusiveness and producing user‐led evidence that aligns with integrated care principles [64]. The findings are likely relevant to other countries, since despite a global movement towards integrated care interventions [65], micro‐level considerations remain limited. The project also encourages cross‐country knowledge mobilization to improve standards of care for people with rare neurological diseases, a population marked by health inequities.

The study excluded non‐English speakers due to budget reasons; despite that limitation, the sample benefited from capturing the experiences of some ethnically diverse participants. There was a risk of interviewer bias since one researcher conducted all the interviews and coded the full data set, although methodological rigour and safeguards were applied: interview guide was co‐designed with PPIE, analysis was led by researchers trained in qualitative methodology, analysis followed the Framework Method [24], and data analysis and interpretation were conducted with PPIE.

## 5 Conclusion

This study developed the first micro‐level integrated care model for people affected by HD. This study's findings are novel since EC4Neuro fills a critical gap, shifting the focus from system structures and institutional processes to a person‐centred approach. This is important for the development and evaluation of integrated care interventions to account for and value its micro‐level impact. Our results highlight the importance of moving beyond healthcare utilization and clinical outcomes to person‐centred outcomes that capture the impact of integrated care programmes on the wider determinants of health.

As a first step, Integrate‐HD provides a foundation for testing whether people‐driven approaches improve care integration [16]. Future work should pilot and evaluate the EC4Neuro model across neurological conditions. Without the adoption of a model of care like EC4Neuro, those most in need of integrated care will continue powerless and marginalized by the system.

## Author Contributions

**Sandra Bartolomeu Pires:** conceptualization, investigation, funding acquisition, writing – original draft, methodology, writing – review and editing, formal analysis, project administration, data curation, resources. **Dorit Kunkel:** conceptualization, funding acquisition, writing – review and editing, validation, methodology, supervision, formal analysis, investigation. **Karine Manera:** writing – review and editing, validation, formal analysis, conceptualization. **Nicholas Goodwin:** writing – review and editing. **Christopher Kipps:** writing – review and editing. **Mari Carmen Portillo:** conceptualization, investigation, funding acquisition, writing – review and editing, validation, methodology, formal analysis, supervision.

## Ethics Statement

This study was approved by the University of Southampton Faculty Ethics Committee (Number 83021).

## Consent

All participants involved in this research provided written informed consent.

## Conflicts of Interest

The authors declare no conflicts of interest.

## Supporting information


**Figure S2:** Venn diagram incorporating the EC4Neuro model components with some of the top ranked person‐centered outcomes, to exemplify correspondence between components and outcomes.

**Table S1:** Integrate‐HD II interviews codebook developed in software NVivo15.

Appendix 1 Supporting information 20260121.

## Data Availability

The interview data are openly available in PURE. The workshops transcripts are not shared for the risk of compromising ethical standards. The workshops ranking exercises are shared in Supporting Information S3: Appendix 1.
