# Supplementary material for: Co‐Design of a New Integrated Care Model With People Affected by Huntington's Disease: A Mixed Methods Study
Source: Health Expect. 2026 Feb 1;29(1):e70584. doi: 10.1111/hex.70584 (PMC12860904; doi:10.1111/hex.70584)
Supplement: Supplementary file 1 — Figure S2: Venn diagram incorporating the EC4Neuro model components with some of the top ranked person‐centered outcomes, to exemplify correspondence between components and outcomes. [file HEX-29-e70584-s003.docx]

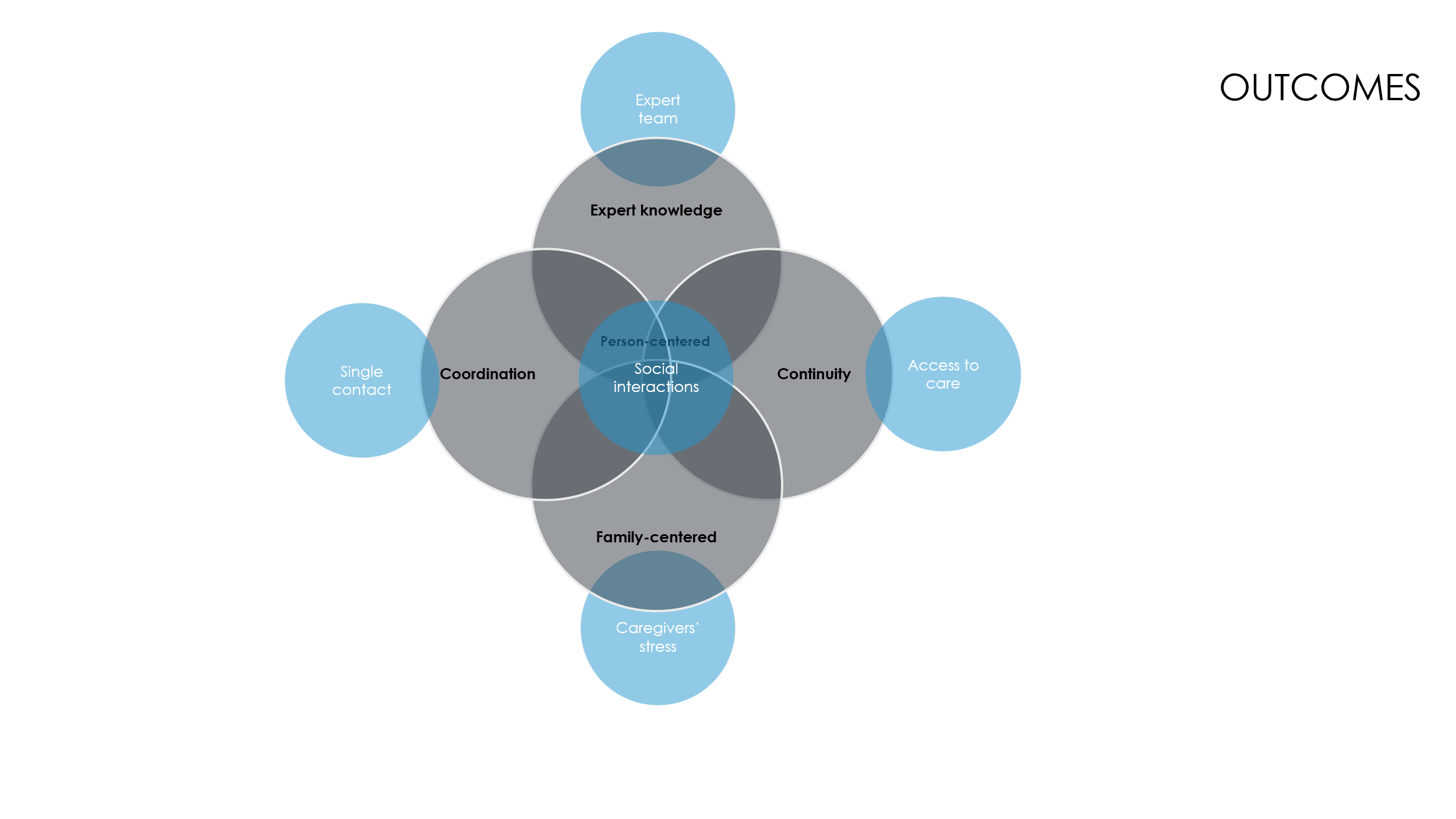


**Figure S2** Venn diagram incorporating the EC4Neuro model components with some of the top ranked person-centered outcomes, to exemplify correspondence between components and outcomes.
