# Supplementary material for: Co‐Design of a New Integrated Care Model With People Affected by Huntington's Disease: A Mixed Methods Study
Source: Health Expect. 2026 Feb 1;29(1):e70584. doi: 10.1111/hex.70584 (PMC12860904; doi:10.1111/hex.70584)
Supplement: Supplementary file 2 — Table S1: Integrate‐HD II interviews codebook developed in software NVivo15. [file HEX-29-e70584-s001.docx]

**Table S1** Integrate-HD II interviews codebook developed in software NVivo15.

| Name | Description | Files | References |
| --- | --- | --- | --- |
| 1_**HD Care characteristics** | How? | 0 | 0 |
| Continuity | Continuity as understood by Freeman • longitudinal or provider continuity – seeing the same professional • continuity across the secondary/primary care interface concerning discharge from specialist to generalist care; supported transitions (CCM-NC) • continuity of information through records – either written or electronic | 23 | 189 |
| Coordination | People work together to understand the patient and carer (National voices definition) ‘Care coordination’ is a person-centered, assessment-based, interdisciplinary approach to integrating health care and social support services in a cost-effective manner in which an individual’s needs and preferences are assessed, a comprehensive care plan is developed, and services are managed and monitored by an evidence-based process which typically involves a designated lead care coordinator. | 25 | 158 |
| Expert knowledge | Professionals’ expert knowledge of HD and its influence in care experiences, knowledge of the care system | 26 | 262 |
| Family-centered care | Care around family-needs, ripple effect related to HD in the family, impact from prior care experiences e.g. older generations | 24 | 90 |
| Person-centered care | Care around individual needs, preferences and wishes. Tailored care. | 24 | 160 |
| zz_Other HD care characteristics | Any other relevant codes | 0 | 0 |
| 2_**HD Care Pathway** | Treatment alongside the disease lifespan | 0 | 0 |
| Asymptomatic stage | Needs and care around people who are not diagnosed with HD, but are at risk or gene positive, prior disease manifests (e.g. predictive test). | 14 | 45 |
| Carers care pathway | Needs and care around informal carers, as partners in care, through living with HD | 18 | 90 |
| Diagnosis stage | Needs and care around diagnostic stage, when disease starts manifesting or there is suspicion (e.g. psychiatric symptoms management) | 14 | 53 |
| Follow-up_Chronic disease stage | Needs and care around the chronic disease stage. Review/Follow-up appointment, referrals, equipment needed | 21 | 168 |
| Research | The role and impact of research in living with HD | 13 | 44 |
| zz_Other care pathways | Any other codes, such as end of life, palliative care, rehabilitation | 5 | 19 |
| 3_**HD burden** |  | 0 | 0 |
| Avoidable burden | The added (avoidable) burden caused by external factors that generates workload for the patient and family, such as care fragmentation leading to chasing services | 0 | 0 |
| (No) Pathway | Not knowing what to expect, no pathway | 18 | 44 |
| Advocacy work | Activities/work to promote HD as a serious health condition | 19 | 69 |
| Care not accessible | Consequences of poor and delayed access to care, reactive care culture | 21 | 76 |
| Own coordination | Activities/work to keep different care providers engaged in care, e.g. chasing professionals | 19 | 68 |
| Own-monitoring_tracking | Responsibility for tracking disease development and take action if changes occur, for themselves or the person they care for | 8 | 14 |
| Unavoidable burden | Burden directly linked with disease signs, symptoms and management. Proper support can help better living with, but not all suffering is avoidable | 0 | 0 |
| At-risk children | Having children at risk | 13 | 39 |
| Family dynamics | HD impact on family relationships, troubles with staying engaged/connected within the family | 19 | 74 |
| Life decisions with HD | Life continues with and despite HD impact. Decisions are aggravated/made harder due to HD, e.g.: insurance access, life-planning (predictive test) | 12 | 32 |
| Psychiatric_Mental health symptoms | Impact of non-motor symptoms, particularly psychiatric changes, on living and caring for people with HD, including safeguarding and risk management | 22 | 163 |
| zz_Other HD burden |  | 0 | 0 |
| 4_**Capacity to manage HD burden** | The “tools” as disease progresses, individual’s capacity goes down, while professional’s capacity needs to go up. Who does the work? Cognitive participation | 0 | 0 |
| Providers' capacity (+-) | The individual and service characteristics that influence care delivery | 0 | 0 |
| Attitudes and beliefs | Professionals’ attitudes towards HD and HD care, existence or absence of working principles such as professional curiosity, collaborative culture, valuing continuity of care | 23 | 194 |
| Morale | Professionals’ morale at work | 11 | 26 |
| Professional resources | Resources available to train providers and have information accessible, e.g. GP knowing who to contact for social care support | 17 | 74 |
| Rapport_Relationships | How different relationships affect care experiences and outcomes, between professionals and professionals-users interactions | 19 | 105 |
| Roles_Agencies | Variety of professionals involved in care and sort of sectors | 24 | 979 |
| Upskilling professionals | For non-expert professionals, how to keep staff trained to care for PwHD | 9 | 41 |
| User's capacity (+-) | The individual’s characteristics that influence living with the disease and different care experiences | 0 | 0 |
| Carer | Carer existence and ability to care | 20 | 203 |
| Comorbidities | Presence, type and impact of comorbidities | 12 | 24 |
| Engagement with services | Capacity to engage with services and attitude towards care and the disease | 19 | 51 |
| Personal_Social networks | Existence, dynamic, impact, e.g. peer support present | 17 | 59 |
| Self-management | Capacity and insight to manage the disease, presence and absence of self-management capacity, use of resources | 21 | 124 |
| User resources | Resources available to empower users and increase health literacy | 15 | 74 |
| Work | Work conditions, individual in working age, financial capacity/support | 13 | 43 |
| zz_Other capacity to manage HD burden | Any other relevant codes | 0 | 0 |
| 5_**Interventions** | Suggestions that can help improve care | 0 | 0 |
| Provider-driven interventions | Care improvements and changes suggested by provider-participants | 9 | 59 |
| User-driven interventions | Care improvements and changes suggested by service users-participants | 13 | 65 |
| zz_Other interventions | Any other relevant codes | 0 | 0 |
| 6_**Outcomes** | What success looks like from people's perspective | 0 | 0 |
| Carer-specific outcomes | The change a carer would feel or be able to see, should care be more integrated. | 15 | 50 |
| Person-centered outcomes | The change people would feel or be able to see, should care be more integrated. | 20 | 72 |
| zz_Other outcomes | Any other relevant codes | 7 | 19 |
| 7_**Factors associated with care integration** |  | 0 | 0 |
| Barriers and Facilitators | barrier to accessing care and promoting change | 0 | 0 |
| Access to care | Delays, waiting list, gatekeepers | 22 | 145 |
| Accountability + | Standards, audits e.g. nursing homes that care for HD patients | 12 | 26 |
| Awareness + | Empathy and understanding of the disease, not just the public but people closer to patient and family | 16 | 41 |
| Bureaucracy | Paperwork and formal processes | 9 | 26 |
| Care settings | Long term care settings not appropriate to people with HD, difficult to find housing | 19 | 114 |
| Carer not involved | Carers not considered as partners in care | 10 | 36 |
| Choice_Control + | Having options, for example, which formal carer to take in. Being able to say yes or no without detriment. Have non-pharmacological therapies offered. Have flexibility | 15 | 43 |
| COVID | Impact from COVID-19 | 13 | 21 |
| Cross-boundaries + | Flexible collaborative working | 13 | 61 |
| Culture and Language | Different cultures, blaming culture, societal values | 16 | 55 |
| Financial | Local commissioning, agencies are funded in a way that promotes fragmentation and silo working, lack of economic investment, reduced funding | 19 | 114 |
| Human resources availability | Reduced service capacity, delayed response | 19 | 106 |
| Interest + | Motivation, curiosity, value | 12 | 32 |
| Low priority | Feelings of abandonment from users, no funding allocated due to low priority label | 9 | 16 |
| Perceived worth(less) | The own person does not see themselves worthy of accessing care. | 5 | 8 |
| Personal relationships Distrust - | Impact of distrust in care settings/relationships | 14 | 41 |
| Personal relationships Trust + |  | 11 | 16 |
| Point of contact + | Single clear point of contact, available and accessible | 19 | 56 |
| Politics_Governance | Lack of policy and politics to protect vulnerable people | 13 | 48 |
| Post code lottery_Health inequalities | Geographical variation affecting care access and quality | 12 | 39 |
| Predictive testing required | Need for a genetic result to access some services, namely mental health services | 2 | 5 |
| Professional competence + | Knowledge, experience | 19 | 97 |
| Professional continuity + | The same professional/team following the user | 15 | 45 |
| Reactive vs proactive care | Not personalized, waits for crisis to occur and then act, instead of investing in preventing crisis | 16 | 39 |
| Sector-focused work_system rigidity_structural barriers | "not with me". There is no easy dynamic for articulating different sectors, particular difficulties with GP and social care (strict boundaries); Difficult cross-boundaries work | 14 | 41 |
| Staff stability | Rotation of staff | 12 | 27 |
| Stigma | Negative feelings, disapproval, bad opinion, disrespect, exclusion | 15 | 41 |
| Technology_teleHealth + | Telehealth more available | 5 | 12 |
| Technology_teleHealth_technical barriers - | Telehealth as a negative, no data centralization, access to medical records | 15 | 51 |
| Time sensitive action needed | The urgent nature of HD and need for a quick response. | 14 | 27 |
| Training offered + | Information and training available to users and providers who need it | 14 | 72 |
| Travelling distances | Geographical barriers related to accessibility | 10 | 17 |
| Unfit assessments | Benefits assessments unfit for purpose, not accessible to the lay citizen | 11 | 19 |
| Voluntary sector lacks autonomy | Voluntary sector with responsibility but without commissioning | 3 | 6 |
| Vulnerability | People's fragility, disempowerment, makes them easy targets for abuse and exploitation | 11 | 22 |
| Wider determinants of health | Housing, heating, transport, social integration/community support, etc. | 8 | 17 |
| Attitudes | positive or negative experience, impact, feeling | 0 | 0 |
| - Negative |  | 25 | 420 |
| + Positive |  | 23 | 359 |
